# Expenditure variations analysis using residuals for identifying high health care utilizers in a state Medicaid program

**DOI:** 10.1186/s12911-019-0870-4

**Published:** 2019-07-12

**Authors:** Chengliang Yang, Chris Delcher, Elizabeth Shenkman, Sanjay Ranka

**Affiliations:** 1 Department of Computer & Information Science & Engineering, University of Florida, Gainesville, FL, USA; 2 Department of Health Outcomes & Biomedical Informatics, University of Florida, Gainesville, FL, USA

**Keywords:** Residual analysis, High utilizers, Preventable cost, Risk adjustment, Regression, Tree-based model

## Abstract

**Background:**

High utilizers receive great attention in health care research because they have a largely disproportionate spending. Existing analyses usually identify high utilizers with an empirical threshold on the number of health care visits or associated expenditures. However, such count-and-cost based criteria might not be best for identifying impactable high utilizers.

**Methods:**

We propose an approach to identify impactable high utilizers using residuals from regression-based health care utilization risk adjustment models to analyze the variations in health care expenditures. We develop linear and tree-based models to best adjust per-member per-month health care cost by clinical and socioeconomic risk factors using a large administrative claims dataset from a state public insurance program.

**Results:**

The risk adjustment models identify a group of patients with high residuals whose demographics and categorization of comorbidities are similar to other patients but who have a significant amount of unexplained health care utilization. Deeper analysis of the essential hypertension cohort and chronic kidney disease cohort shows these variations in expenditures could be within individual ICD-9-CM codes and from different mixtures of ICD-9-CM codes. Additionally, correlation analysis with 3M™ Potentially Preventable Events (PPE) software shows that a portion of this utilization may be preventable. In addition, the high utilizers persist from year to year.

**Conclusions:**

After risk adjustment, patients with higher than expected expenditures (high residuals) are associated with more potentially preventable events. These residuals are temporally consistent and hence may be useful in identifying and intervening impactable high utilizers.

## Background

The Agency for Healthcare Research and Quality (AHRQ) reports that in 2012, the top 10% of the health care-utilizing population accounted for 66% of overall health care expenditures in the United States [1]. This highly disproportionate spending pattern frequently is interpreted as a sign of inefficient health care delivery and partially associated with avoidable, preventable or otherwise unnecessary health care events. Nationally, in 2010, potentially avoidable emergency department (ED) encounters accounted for $64.4 billion, 19.6% of ED episodes, and 2.4% of national health expenditures [2]. In this context, stakeholders have argued the need for higher efficiency health care for such patients, sometimes referred to as “high utilizers” [3]. For example, the deployment of managed care organizations (MCOs) and the capitation payments system [4] in the United States public health programs provide incentives for health care providers to deliver services in a more cost-effective way.

The Centers for Medicare and Medicaid Services (CMS) recommends [5] that state Medicaid programs determine the extent to which expenditures driven by high utilizing populations represent “impactable” costs. Therefore, it seems reasonable to examine the avoidable health care conditions in this population, as these events may be more amenable to prevention and cost reduction strategies [6]. As an example at the state-level, the Minnesota Department of Public Health found that in 2012, nearly 1.3 million visits to hospitals in the state, representing nearly $2 billion in associated costs, could have been potentially prevented [7].

Regional disparities [8] is shown to be important for health care expenditure variations in the United States. At the individual level, several existing studies [9–11] identify high utilizers based on the total number of visits or total expenditures per unit time or some combination thereof. Existing studies find that historic utilization is indicative for future utilization [12]. While using such data-driven methods may be a relatively straightforward starting point for Medicaid programs with limited analytic resources, the approach is blunt and may fail to identify patient populations with health conditions most responsive to prevention and, by extension, cost reduction. To illustrate one problem of relying on count-and-cost based criteria alone, consider that during flu season, increased visits for uncomplicated respiratory illnesses may contribute to temporary increases in ED overcrowding without necessarily resulting in high-cost medical treatments [13]. Furthermore, patients with serious conditions, such as cancer or traumatic injuries, may require expensive medical treatments that seem excessive as financial data points but that are medically appropriate and necessary.

An alternative strategy may involve more sensitive data-driven approaches that identify more impactable subpopulations of high utilizers even though they may not incur the highest absolute expenditures. We assume that this group of patients is “over-utilizing” the health care system because they spend more than their expected costs based on adjustments for health care-utilizing factors. Though clinical diagnostic classification tools such as the New York University ED profiling algorithm [14] and 3M™ Potentially Preventable Events (PPE) [15] software are designed to improve the ability to identify preventable or avoidable conditions, they have a number of limitations, including a limited population scope, lack of transparency due to commercial considerations, and others.

This paper proposes an approach to address such limitations. Briefly, using insurance claims data typically available to state Medicaid programs, our data-driven approach calculates expenditure expectations for specific health care conditions and other measurable factors first; then we identify patients whose expenditures are higher than expected (i.e. presumed overutilization). The magnitude of overutilization is quantified by the degree to which cost residuals deviate from the model for each patient. Thus, patients with higher-than-expected residuals represent a population of health care utilizers with health care costs that may be inexplicably excessive and potentially associated with a nontrivial fraction of preventable conditions. Additionally, we compare the performance of two regression approaches, one standard and one based on decision trees ensembles used more frequently in computer science, to make recommendations regarding the growing interest in applying data-driven methods in the biomedical sciences.

Briefly, the key contributions of our paper are as follows:

- We identify a best-fitting linear and tree-based regression model to account for patients’ acute and chronic conditions loads and demographic characteristics using a large administrative claims dataset spanning 4 years from a public insurance program with millions of patients.
- We identify populations with the highest deviations from expected costs after adjustment and examine their characteristics. Furthermore, we examine whether the model identifies the same set of patients consistently through time. The results suggest that our approach identifies a significant proportion of health care costs, which persists from year to year.
- We examine the variations of expenditures associated with two medical diagnoses: hypertension and chronic kidney disease. We find significant variations in expenditures within some diagnoses.
- We stratify the model by two health care service settings, the inpatient acute care hospital and emergency department. In each setting, we compare our results identifying potentially preventable conditions with an existing commercial clinical tool, the 3M™ PPE software.

The rest of this paper is organized as follows: In “Methods” section, we introduce the dataset and methods used for the study. Specifically, we describe the risk adjustment models and define how residuals are used to identify high utilizers. “Results” section presents the results, including examining the demographics, clinical characteristics, utilization profiles and temporal consistency of the high-utilizing population, and comparing the residuals approach with the 3M™ PPE tool. “Discussion” section presents conclusions and discussion. A preliminary version of this work has been reported [6].

## Methods

### Study population

In this study, we use an administrative claims and encounter dataset from the Medicaid insurance program of Texas. We set the inclusion criteria at a yearly basis. For each year from 2011 through 2014, we include adult (18–60 years old) Texas Medicaid beneficiaries, excluding pregnant women, with nonzero expenditures. The resulting size of the study population for 2011 to 2014 is 464,572, 530,242, 514,601, and 535,423 respectively.

### Data preprocessing

To preprocess the dependent variable, we normalize the health care expenditures to a per-member per-month dollar amount (total medical expenditure divided by number of months enrolled in Medicaid). This measure is commonly used for expenditure analyses in Medicaid programs [16] and, given that individuals may enroll in Medicaid for differing lengths of time, adjusts for variation in time enrolled.

For the independent variables, to more meaningfully summarize the 21,374 unique diagnosis codes (International Classification of Diseases, Ninth Revision, Clinical Modification, ICD-9-CM) identified from the dataset, first we group ICD-9-CM codes into 283 categories using AHRQ’s Clinical Classification Software [17]. Then we transform all categorical variables to one-hot encoding. All the variables included in the models are presented in Table 1.

**Table 1:** Variables Specification

Dependent Variable	Per-member per-month expenditure
Independent Variables	Disease categories: ICD-9-CM codes grouped into Clinical Classification Software categories (CCS) [17]
	Demographics: age, sex, race, and disabled status
	Geographical information: county of residence
	Health insurance programs and plans: fee-for-service, managed care organization plans

### Model

Two types of statistical models, linear regression and tree-based model, are adopted to adjust the risk factors (independent variables) for health care expenditures.

#### Linear regression

Linear regression based adjustment models [4] have been used in health care capitation payments as they systematically account for spending associated with specific health care conditions. Generally, we can write the model into the equation below:

1 $$ y = \beta \mathbf{x} + \epsilon   $$

where *y*, **x**, and *β* represent expenditure, a vector of exogenous health care utilization factors and their linear coefficients respectively. If all the factors associated with health-care expenditures are exogenous, inclusion of these factors would perfectly explain all the variations in health care expenditures except for an independent, normally distributed error *ε*. If this is the case, the residuals, which are the observed error term *ε* in (1), should follow the normal distribution. This is also an assumption of classical linear regression. If the residuals have a longer right tail than the normal distribution, we would expect that the model does not account for unmeasured factors. We set up the linear regression as specified in Table 1 to account for the patients’ health care conditions, demographics and health insurance plan differences. Note that we adjust for only the exogenous variables largely considered non-modifiable, such as health conditions, demographics, residence county, etc., but not for the endogenous variables like frequency of health care visits.

We use ordinary least squares (OLS) to find the best fit for the linear regression health care utilization adjustment model.

#### Tree-based model

Though linear regression is widely used for risk adjustment because of the relative ease of use, interpretability and well-established statistical properties, we note that it cannot easily capture interactions between independent variables. For example, traditional epidemiologic linear regression models rarely include more than three interaction terms. Given that we have hundreds of disease categories in our model and the interactions between them represent complex, potentially non-linearly related comorbidities that might greatly influence the health care needs of the patients, we may attempt to include these interactions in the risk adjustment. However, due to the high dimensionality of the independent variables, it is impractical to add all possible interactions to the linear regression model. Thus, we examine a decision tree-based model as an alternative to the linear regression model because tree-based models [18] can capture N-way interactions automatically. Tree-based models have been used to study hospital readmissions [19] and classification of epilepsy patients [20].

Gradient boosting machine (GBM) [21] is one of the most powerful tree-based models to handle high dimensional datasets. The model learns an ensemble of decision trees *f*_*t*_ in an additive manner. In each round, it adds a new tree *f*_*t*_ to the model by optimizing the objective function of:

2 $$ \min_{f_{t}}\sum\limits_{i=1}^{M}(g_{i}f_{t}(\mathbf{x}_{i})+\frac{1}{2}h_{i}f_{t}^{2}(\mathbf{x}_{i}))+\gamma T+\lambda\sum\limits_{j=1}^{T}w_{j}^{2}  $$

where *g*_*i*_ and *h*_*i*_ are the first- and second-order derivatives (gradient and Hessian) of the loss function. In our case, the loss function is the squared error between predicted expenditures $\hat {y}$ and true expenditures *y*, i.e., $|\hat {y}-y|^{2}$. To control mode complexity, the last two terms are added as regularizers, which penalize over-complicated models. *T* is the number of decision tree leaves of *f*_*t*_, and *w*_*j*_ are the leaf weights.

Unlike linear regression, the gradient boosting machine and other tree-based models are not well established for statistical properties, such as the expectation of independently normally distributed error *ε*. Thus we are not able to conduct equivalent statistical tests of normality for the residuals. However, comparison with linear regression is addressed.

### Fitting the model

In this section, we describe the process to fit the linear regression model and the gradient boosting machine model to adjust the risk factors that account for health care expenditures.

#### Fitting linear regression

**Fig. 1:**
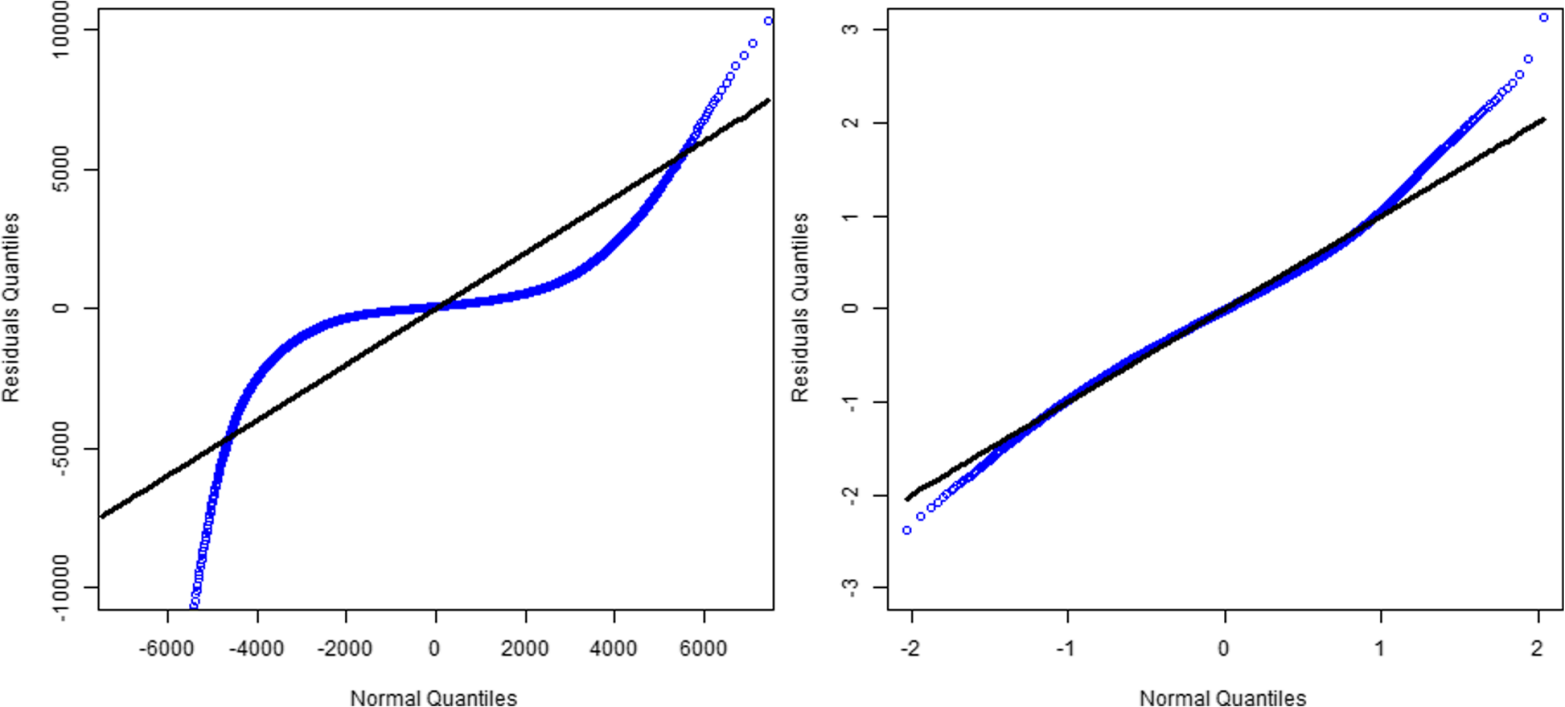
Quantile-to-quantile plot (Q-Q plot) of standard normal distribution and residuals obtained from the linear regression model for year 2014. In the left panel, the dependent variable, per-member per-month expenditures, is not transformed. Due to its strong skewness, the residuals significantly deviate from normal distribution. In the right panel, we log-transform the dependent variable with base 10 and re-run the model. The residuals distribution better fits a normal distribution. The right and left ends are heavy-tailed, indicating over/under utilization

As described, we use the ordinary least squares (OLS) procedure to fit the linear regression model. The models are highly significant for each year from 2011 to 2014 (*p*<0.001 for the *F*−*test* of model fit). However, our visual diagnostic checks identify significant skewness and the residuals clearly deviate from normality (Fig. 1). To address these deviations, we log (10)-transform the dependent variable. Consequently, normality improves, as shown in Fig. 1, but deviations remain. Additionally, the R-squared of the model improves from 0.27 to 0.57 on average. Thus, we use the log-transformed dependent variable as the default setting for all models, including the gradient boosting model. As we can see, the log-transformed model shows deviation on the right-hand side, identifying patients with higher-than-expected cost values. In this subpopulation of patients, we hypothesize that a large proportion of potentially avoidable conditions exist that would be suitable for consideration as a potentially “impactful” intervention.

Although multi-collinearity is detected among the independent variables of the model, which is expected based on the large number of variables present, we retain all correlated variables in the model for the following reasons. First, our goal is not to interpret any individual independent variable or estimate its effects. Second, from the adjustment point of view, as long as the independent variables are not completely linearly correlated, we deem them acceptable for inclusion.

**Fig. 2:**
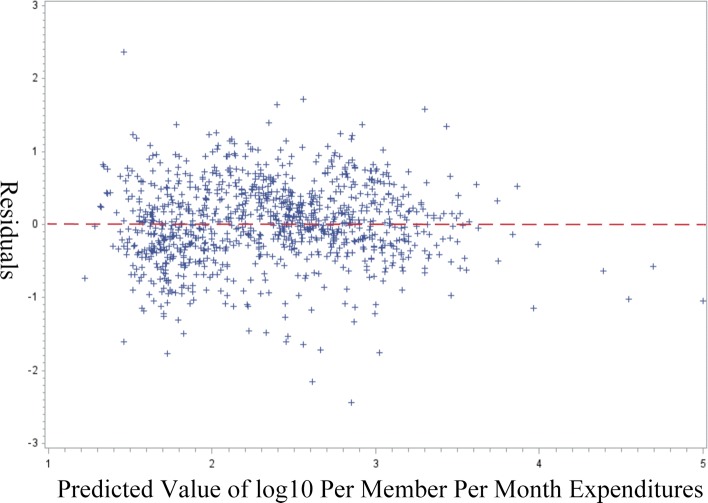
Predicted versus residuals plot for 2000 observation random sample for 2014. No obvious heteroscedasticity is observed from the plot

Heteroscedasticity also could affect the distribution of residuals. If strong heteroscedasticity exists, the high residuals could be those with error terms *ε* of larger variances. We investigate the residuals versus predicted plot (Fig. 2) and find that the residuals are distributed with similar variances. Although the Breuch Pagan test does suggest that heteroscedasticity exists (*p*<0.05), after we adopt the heteroscedasticity-consistent standard errors and re-run the model, the model remains statistically significant. The generalized Gamma model (GGM) provides unbiased estimates [22] if there is heteroscedasticity in the log scale error. However, interpreting the estimates is not the focus of this study. Therefore, we continue with the simpler log transformation approach to handle the skew distribution of expenditures.

#### Fitting tree-based model

To fit our tree-based model, we use the implementation of Xgboost [23], which is efficient and widely-used. We train 1000 decision trees as an ensemble for each model. To select parameters such as maximum tree depth and minimum data samples in the leaf, we adopt a 5-fold cross-validation and select the model with minimum mean squared error.

As previously mentioned, the gradient boosting machine model does not have established statistical tests to examine model fit characteristics. Instead, we use a held-out testing dataset to ensure the model does not overfit, which is a common practice in machine learning [24]. In summary, each time we train a model, we randomly hold out 40% of the data as the testing dataset that will not be included during training. The remaining 60% is the training dataset and is used to fit the model. After training the model, performance measures such as R-squared are calculated on both datasets. In our case, if the R-squared on the training dataset and testing data are similar, we regard the trained GBM model as an adequate and robust model for risk adjustment.

### Identifying the high residuals population

**Fig. 3:**
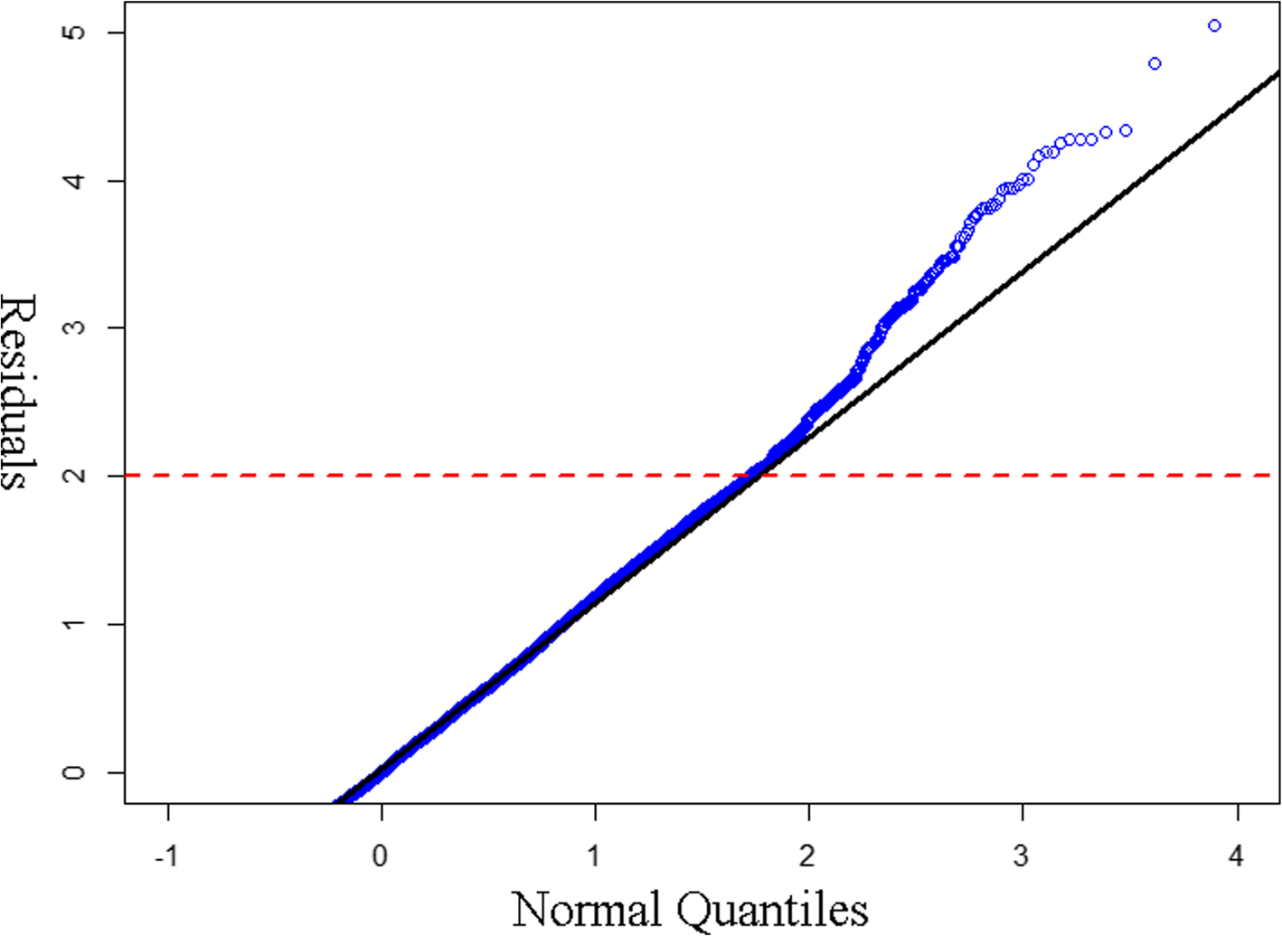
Decide threshold of high utilizer from Q-Q plot: The red dashed line indicates where the long right tail of residuals consistently deviates from the normal distribution quantiles

Visual inspection is clear but we define an empirical threshold to formally discriminate the point at which the right long tail of residuals consistently deviates from the normal distribution. The process is described graphically on the Q-Q plot in Fig. 3. The size of the high utilizer population varies from 1% to 7% of the overall population for different years and model parameter settings. In terms of population size identified, the proportion is similar to count-based methods [25].

To examine the population profiles, we compare the high utilizer group to other patients using demographics, utilization patterns including expenditures of different service categories, and overall health condition burden. Further, to examine the temporal consistency of this spending, which addresses a debate regarding the longer-term occurrence of unusually high spending patterns [10], we use the following two-step process to test the Pearson product-moment correlation between the percentile rank in years from 2011 through 2014.**Step 1:** Rank order all the patients in the index year and and subsequent years based on the absolute value of the residual.**Step 2:** Compute the Pearson product-moment correlation coefficient between the rank percentiles for any year-to-year pairs.

### Breakdown residuals

In our models, available disease information is included using the AQRO’s Clinical Classification Software [17] which consists of a grouping of the original ICD-9-CM diagnosis codes. We can break down the residuals to the ICD-9-CM code-level to see how variations are distributed across different ICD-9-CM codes. To be specific, we choose one disease category every time to check the variations within this disease category. Disease categories are defined by the first three digits of the ICD-9-CM codes, i.e., ICD-9-CM 585xx is chronic kidney disease. Then we follow a 3-step process to show the variation.**Step 1:** For each claim whose principal ICD-9-CM diagnosis code is in the selected disease category, we associate the expenditure of the claim to the principal ICD-9-CM code.**Step 2:** We assign patients to groups of 5000 based on their residuals from high to low.**Step 3:** For each patients group, we show the average per-member per-month expenditures associated with the disease category. In addition, for each patient group and each single ICD-9-CM code in the disease category, we show the number of patients and average per-member per-month expenditures associated with the ICD-9-CM code.

### Stratified model

Inpatient acute care hospital and emergency department (ED) are two important types of health care services. Because inpatient acute care hospital costs are generally an order of magnitude higher than ED costs [25], to better analyze the variations in these two types of services, we stratify the adjustment model by these settings. For this purpose, in each setting, the study population is restricted to those who had nonzero expenditures in the corresponding setting. In addition, we construct dependent variables based only on the setting in which the expenditure occurred. Otherwise, our modeling approaches are the same as above. We compare our results with 3M™ PPE [15] software as a quasi-validation tool. To be specific, we examine if there are differences in PPE measures between high residuals patients and all others. Also, we compute the Pearson correlation coefficient between PPE measures and residuals. Additionally, we test whether the residuals are predictive for future PPEs.

## Results

In this section, we first compare the model-fitting results of the linear regression model and the tree-based model. Then we look at the characteristics and temporal consistency of high utilizers identified by the models. Breakdown of variations in expenditures is examined at the ICD-9-CM code level. Finally, we present the stratified risk adjustment model where we cross-validate the preventability of identified high utilization with 3M™ Potentially Preventable Events (PPE) software.

### Compare linear regression and tree-based model

The linear regression models are highly significant for each year from 2011 to 2014 (*p*<0.001 for the *F*−*test*). The tree-based model (gradient boosting machine, GBM) also appears to fit well to the data based on the minimal differences between the training and testing models. R-squared statistics for both models are presented in Table 2. The GBM model does not appear to overfit, and increases R-squared relative to the linear model, likely due to increased number of interaction terms entered in the model.

**Table 2:** The R-squared statistics calculated from linear regression and gradient boosting machine from 2011 to 2014

R-squared	Linear Regression	Gradient Boosting Machine (GBM)		
		Training	Testing	Overall
**2011**	0.559	0.642	0.628	0.636
**2012**	0.576	0.657	0.646	0.652
**2013**	0.574	0.657	0.645	0.652
**2014**	0.569	0.650	0.641	0.646

**Fig. 4:**
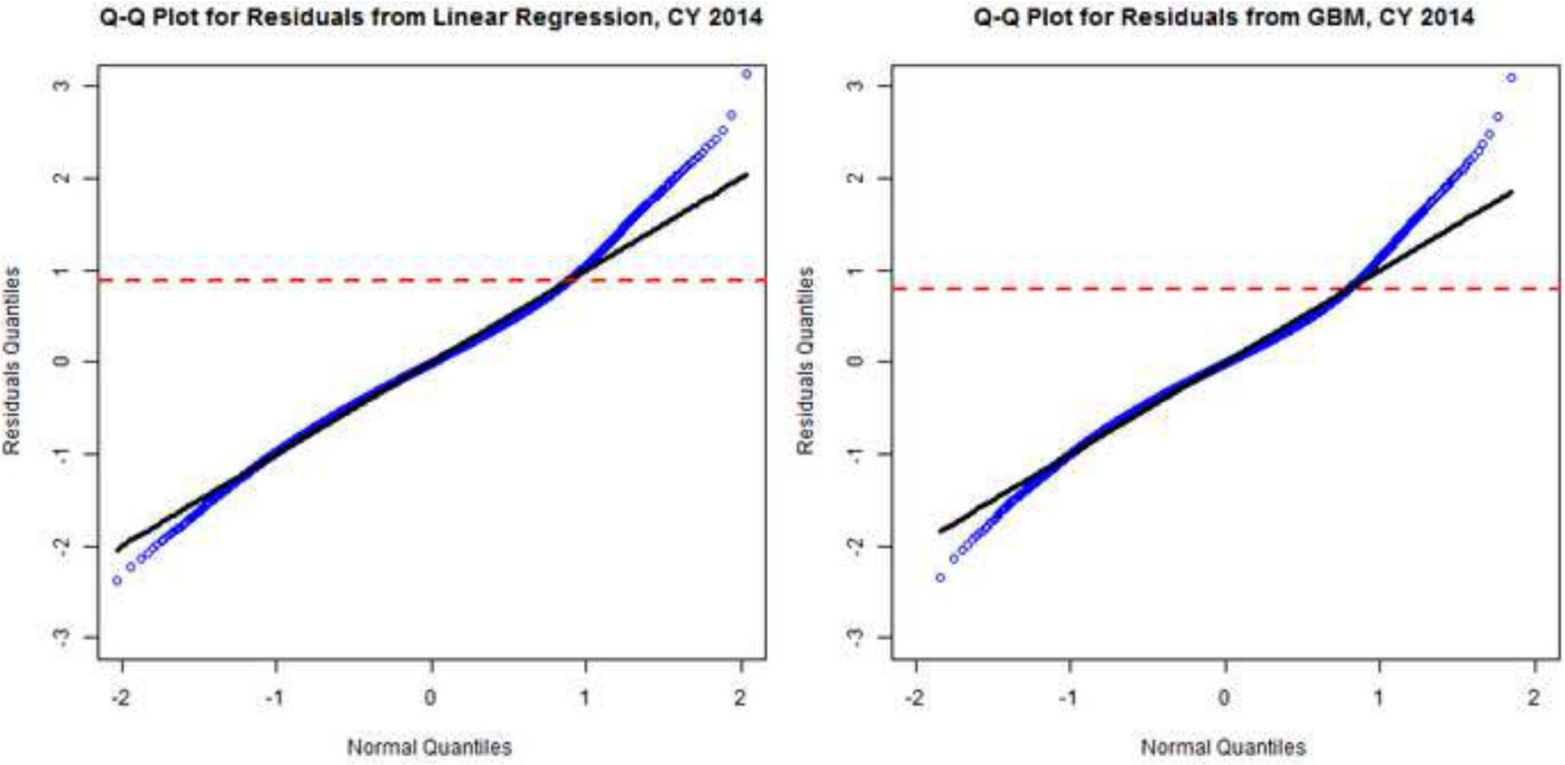
Q-Q plot for the linear regression model (left) and gradient boosting machine model (right) for CY2014. The residuals from both model have long right tail larger than a normal distribution

As our goal is to use the residuals with high variance produced by the models to identify potentially impactful populations, therefore we compare the models in this context. Table 3 shows that the two sets of residuals obtained from both models are highly correlated. In Fig. 4, both linear regression and GBM identify a long right tail larger than a normal distribution. The size of the long right tail varies depending on the model and year of data. To compare the high utilizers identified from the two models, we set a top 5% cutoff threshold and examine the overlap. As shown in Table 4, more than 70% of the top 5% high residuals population identified from linear regression and GBM, respectively, overlap with each other. To summarize, though GBM has a better fit than linear regression does, both models generate highly correlated residuals and identify similar high utilizers. Given what are arguably marginal improvements in R-squared for a complex model that would be more difficult to interpret and implement in practice, we will present results and discussion based on the linear regression model (the model).

**Table 3:** The Pearson correlation coefficient between residuals obtained from linear regression model and GBM model respectively from 2011 to 2014

Year	Correlation Coefficient
**2011**	0.934
**2012**	0.932
**2013**	0.931
**2014**	0.933

The correlation is very high

**Table 4:** The top 5% population with high residuals identified from linear regression and GBM respectively overlap more than 70%

Year	Top 5%, N	Overlap, N	Overlap, %
2011	23,228	16,794	72.3%
2012	26,512	19,119	72.1%
2013	25,730	18,483	71.8%
2014	26,771	19,345	72.3%

### Characterizing the high utilizers

In this section, we take a closer look at the high residuals population. We first descriptively show their characteristics and then study their temporal consistency.

#### Demographics, health conditions, and utilization

We summarize the demographics, health conditions, and health care utilization of high utilizers (top 5% of residuals) and other patients for 2014 in Table 5. These results do not vary significantly by year. In terms of demographics, the high-utilizer group has fewer female patients. Age and race/ethnicity are similar. We use an integrated score, Charlson Comorbidity Index [26], to represent patients’ overall chronic disease burden. The two populations vary little on this measure, which means the categorization of comorbidities is similar. The proportion of mental illness and substance use patients in high utilizers is slightly less than the rest of the population. Thus, we argue that there are no significant differences in demographics and overall health conditions between the high residuals population and the rest. This is not surprising because we expect the model to account for all these factors.

**Table 5:** Comparison of demographics, health conditions and health care utilization of high utilizers (top 5%) with other patients, 2014

Characteristics	High Utilizers (High Residuals)	Other Patients
Number of Patients	26,771	508,652
Demographics		
Mean age (years)	34.11	35.22
Sex		
*Female*, %	58.49	68.47
Race		
*White, non-Hispanic*, %	23.22	26.44
*Black, non-Hispanic*, %	19.04	21.82
*Hispanic*, %	40.10	37.39
*American Indian or Alaskan*, %	0.18	0.19
*Asian, Pacific Islander*, %	2.08	1.57
*Unknown/Other*, %	15.38	12.60
Disabled, %	51.45	45.32
Health Conditions		
Charlson Comorbidity Index [26]	1.66	1.61
Mental illness [9], %	41.49	48.04
Substance use [9], %	29.15	34.00
Utilization		
Average total medical expenditures, $	19,531.87	6588.32
Average professional expenditures^*†*^, $	4937.37	2184.88
Average institutional expenditures^*‡*^, $	7215.62	2450.32
Average pharmacy expenditures, $	7338.35	1940.62
Average number of emergency department visits	0.47	1.03
Average number of inpatient hospital visits	0.38	0.20

^*†*^Professional expenditures represent paid claims generated for work performed by physicians, suppliers, and other non-institutional providers for all medical services

^*‡*^Institutional expenditures represent paid claims generated for work performed by hospitals, skilled nursing facilities, and other institutions for all medical services

However, the levels of health care utilization in the two groups are completely different. In general, the high utilizer group has annual expenditures about three times higher than other patients, and they consistently have more expenditures in each service category. The only exception is that high utilizers visit the ED less frequently than the other patients do. But because ED visits comprise a small fraction of high utilizers’ overall costs, ED expenses have less impact on the overall cost. In a later section, our stratified models examine costs incurred in the ED more specifically.

The findings in this section support our hypothesis that a group of patients whose demographics and comorbidities look similar to other patients has a significantly higher amount of unexplained health care utilization.

#### Temporal consistency of residuals

**Fig. 5:**
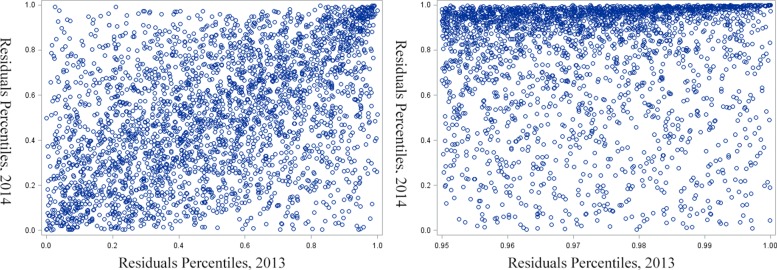
Scatter plot of rank percentiles of residuals: 2013 and 2014. On the left is the scatter plot for overall population. We can visually recognize the temporal correlation as the scatter points are denser on the diagonal from lower left to upper right. On the right is the scatter plot for high utilizers. The temporal correlation for the high utilizers is even stronger than for other patients. Most patients with high residuals in 2013 (top 5%) remain in the high residuals population in 2014 (top of the scatter plot)

Next, we examine the extent to which this excessive utilization persists through time. Generally, if high unexplained variance occurs at random from year to year, then preventing such health care events would be extremely challenging. Otherwise, non-random correlation from year to year may suggest the persistence of health care-utilization factors that might be discoverable. To examine, we compute the Pearson product-moment correlation coefficient between the rank percentiles of residuals for any two subsequent years from 2011 to 2014, as shown in Table 6. Results show significant correlation between residuals from year to year. As expected, the correlation is strongest to the immediate subsequent year and decreases with time. The strong, significant correlation is visually well-represented as a dense diagonal on the scatter plot of rank percentiles of residuals of 2013 and 2014 shown in Fig. 5. We observe an even stronger consistency for the high utilizers (top 5%) because the upper right area is denser. To be specific, the high utilizers of 2011, 2012 and 2013 on average rank 72.5%, 76.3% and 77.8% respectively in residuals percentile for the next year. In comparison, other patients has an average rank of around 50%.

**Table 6:** The Pearson correlation coefficient between the rank percentiles of residuals for any two subsequent years from 2011 to 2014

Year/Correlation	2012	2013	2014
2011	0.396	0.326	0.287
2012	-	0.457	0.380
2013	-	-	0.472

This correlation structure indicates that residuals do not vary randomly from year to year and implies that unobserved factors drive high utilization. In conclusion, the subpopulation corresponding to the high residuals shows an excessive amount of utilization that is temporally consistent.

### Breakdown residuals to ICD-9-CM codes

In this section we analyze the variations in expenditures in more detail, as described in “Breakdown residuals” section. For the essential hypertension cohort (defined by ICD-9-CM 401xx) and chronic kidney disease cohort (defined by ICD-9-CM 585xx), we rank the patients by their residuals from high to low and show the expenditures associated with each single ICD-9-CM code in the cohorts across the residuals spectrum.

#### Essential hypertension

**Fig. 6:**
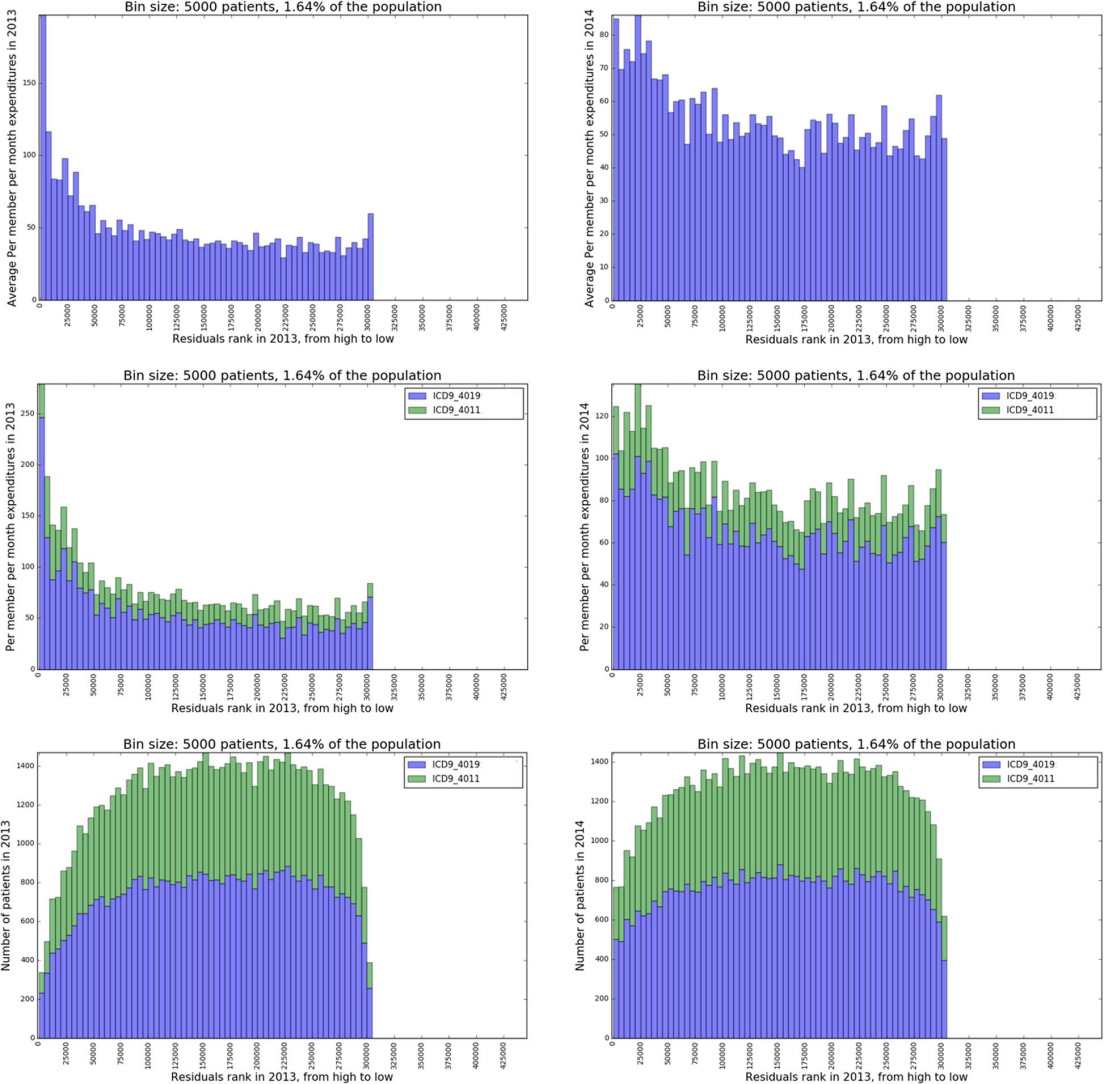
**Row 1:** Overall expenditures associated with essential hypertension (ICD-9-CM 401xx) in 2013 (left) and 2014 (right) respectively by 2013 residuals rank; **Row 2:** Breakdown of essential hypertension expenditures by associating with each single ICD-9-CM code in 2013 (left) and 2014 (right) respectively by 2013 residuals rank; **Row 3:** Number of patients associated with each single essential hypertension diagnosis ICD-9-CM code in 2013 (left) and 2014 (right) respectively by 2013 residuals rank

Figure 6 breaks down expenditures associated with essential hypertension and show how it varies across the residuals spectrum. It first shows in the hypertension cohort, patients with higher residuals in 2013 spend much more on hypertension (primary diagnosis is hypertension) than other groups. This difference continues to 2014. After breaking down the hypertension expenditures by ICD-9-CM codes (using primary diagnosis), we find that within the diagnosis ICD-9-CM 4019 (Unspecified essential hypertension) there is significant variation, which is driving the variation of the overall hypertension expenditures. Finally it is shown that the proportion of the two types of hypertension ICD-9-CM codes does not change much across the residuals spectrum. So the proportion of patients associated with these two ICD-9-CM codes is not the primary driving factor of the variation of overall hypertension expenditures. To conclude, the major source of variation of essential hypertension expenditures is within ICD-9-CM code 4019 (Unspecified essential hypertension).

#### Chronic kidney disease

**Fig. 7:**
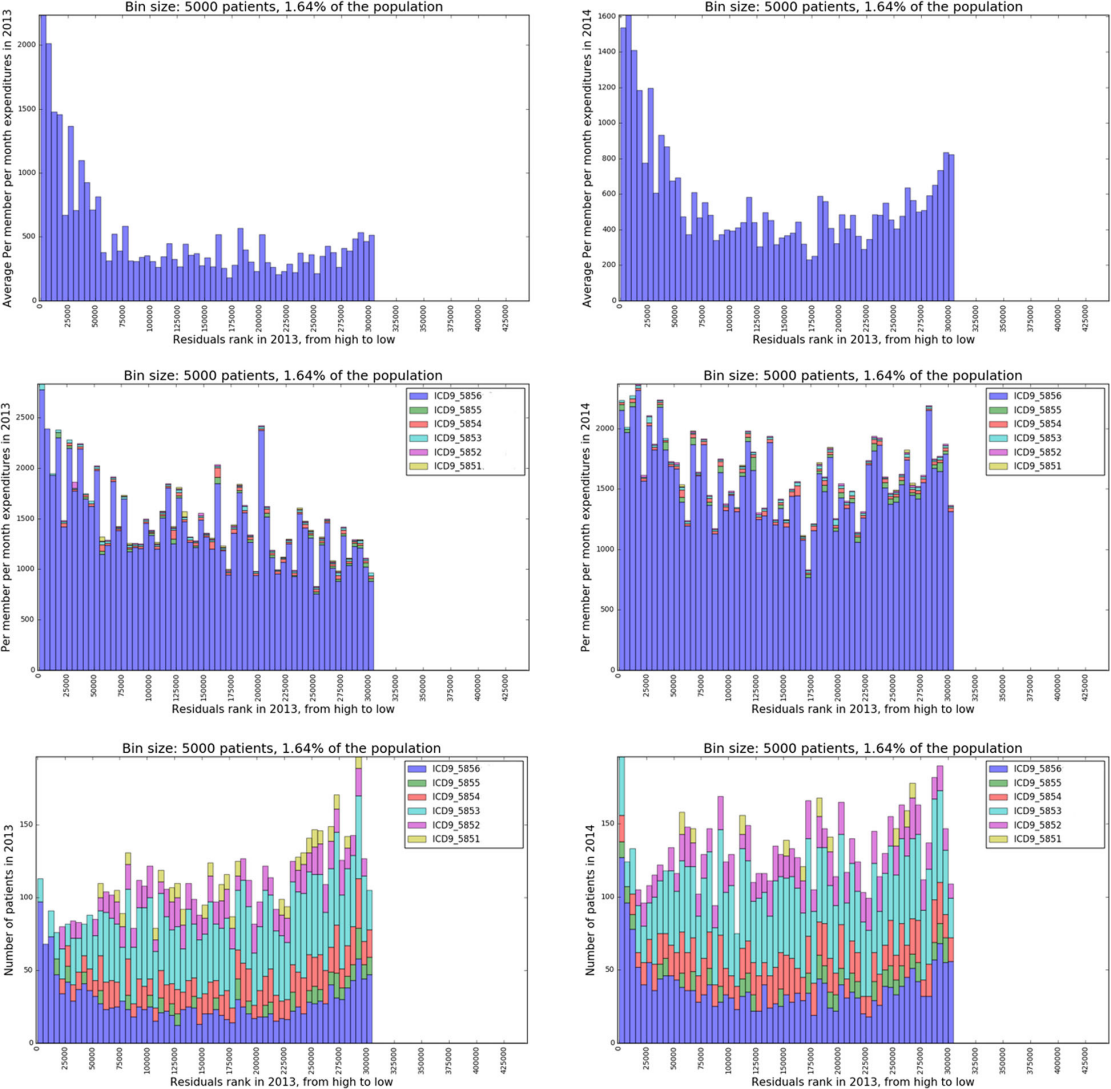
**Row 1:** Overall expenditures associated with chronic kidney disease (ICD-9-CM 585xx) in 2013 (left) and 2014 (right) respectively by 2013 residuals rank; **Row 2:** Breakdown of chronic kidney disease expenditures by associating with each single ICD-9-CM code in 2013 (left) and 2014 (right) respectively by 2013 residuals rank; **Row 3:** Number of patients associated with each single chronic kidney disease ICD-9-CM diagnosis code in 2013 (left) and 2014 (right) respectively by 2013 residuals rank

The same analysis above is conducted on the chronic kidney disease cohort. The results are presented in Fig. 7. As hypertension, high residuals groups are higher cost groups of patients with chronic kidney disease. The variation persists from 2013 to 2014. After breaking down the chronic kidney disease expenditures by ICD-9-CM codes, we find that ICD-9-CM 5856 (End-stage renal disease) is the main driver of the expenditures. However, as shown in Fig. 7, the variation within this specific diagnosis is not as large as the variation of overall expenditures of chronic kidney disease. It shows that the proportion of patients with end-stage renal disease (ICD-9-CM 5856) and earlier stages of renal disease (ICD9-5851 to ICD9-5855) is very different across the residuals spectrum. The high residuals end contains more patients with end-stage renal disease (ICD-9-CM 5856), and the lower end of the spectrum has fewer of these patients. So the variation of the overall expenditures is from different mixtures of patients with different ICD-9-CM codes of chronic kidney disease.

Overall, the results show that there are variations of expenditures within individual ICD-9-CM codes. Different mixtures of ICD-9-CM codes could drive variations as well. We might be more interested in the first type of variation because we could assume the same diagnosis code should induce similar costs. Further medical records review could help reveal the source of these types of variations.

### Stratified models by service settings

We have shown that the residuals indicate an unexplained high amount of health care utilization. Next, we examine whether this utilization is associated with preventable costs. The 3M™ PPE software [15] identifies potentially preventable health care events in the inpatient hospital and ED settings. The software is proprietary but its validity has been verified and widely accepted [27]. In this section, we examine the correlation between the 3M™ PPE approach and our own residuals approach. 3M™ Potentially Preventable Readmissions (PPR) Grouping Software identifies clinically related and potentially preventable inpatient hospital readmissions. 3M™ Potentially Preventable Emergency Visits (PPV) Grouping Software identifies ED visits that relate to ambulatory-sensitive conditions and may result from lack of access to primary care. Thus, we can identify potentially preventable utilization in two important service settings: inpatient hospital and ED. We modify the dependent variable to reflect expenditures incurred only in those settings and re-run the linear regression risk adjustment model. Independent variables are kept the same. By comparing the PPE statistics and resulting residuals from the stratified model, we examine our hypothesis that our approach may show similar performance.

#### Residuals and Potentially Preventable Readmissions (PPR)

Table 7 presents the PPR statistics for the high utilizer group versus other patients. High utilizers are identified using the approach described in “Identifying the high residuals population” section and comprise usually 3–4% of the population. The high-utilizer group has a significantly (Mann–Whitney U test, *p*<0.05) higher amount of PPR events and PPR expenditures than all others. The difference is consistent from 2011 to 2014. This suggests that the high residuals are associated with a substantial amount of potentially preventable hospitalizations. The Pearson correlation coefficients between the residuals and PPR expenditures tell the same story. PPR expenditures are log-transformed to scale with the residuals. We compute the correlation coefficient with the residuals obtained from the stratified model using inpatient hospital expenditures as the dependent variable, as shown in Table 8. The correlation is always significant through the years, suggesting a strong association between residuals and PPRs.

**Table 7:** Potentially Preventable Readmissions (PPR) statistics of index year and next year for high utilizers versus other patients

	Index year	High Utilizers (High Residuals)	Other Patients
Average number of PPRs in index year	2011	1.24	0.15
	2012	1.12	0.20
	2013	1.10	0.21
	2014	0.86	0.19
Average PPR expenditures in index year, $	2011	7793.23	1200.63
	2012	6807.52	1259.59
	2013	6229.76	1237.03
	2014	5241.54	1112.03
Average number of PPRs in next year	2011	0.70	0.17
	2012	0.50	0.18
	2013	0.37	0.14
Average PPR expenditures in next year, $	2011	3698.67	1113.45
	2012	2549.41	1001.92
	2013	2072.29	758.82

Residuals and high utilizers are identified from index year. Per-member per-month inpatient hospital expenditures is the dependent variable of the model

**Table 8:** The Pearson correlation coefficient between the residuals from the stratified model (inpatient hospital and emergency department) with Potentially Preventable Readmissions (PPR) expenditures and Potentially Preventable Emergency Department Visits (PPV) expenditures respectively from 2011 to 2014

	Index Year	Correlation Coefficient with Residuals
*log*_10_(Per-member per-month PPR expenditures of index year)	2011	0.2974
	2012	0.2638
	2013	0.2755
	2014	0.2664
*log*_10_((Per-member per-month PPV expenditures of index year)	2011	0.2452
	2012	0.3214
	2013	0.3423
	2014	0.3165
*log*_10_(Per-member per-month PPR expenditures of next year)	2011	0.0990
	2012	0.0972
	2013	0.0948
*log*_10_((Per-member per-month PPV expenditures of next year)	2011	0.0908
	2012	0.1051
	2013	0.1121

Residuals and high utilizers are identified from index year

#### Residuals and Potentially Preventable Emergency Department Visits (PPV)

We replicate the analysis for potentially preventable ED visits. The population is limited to patients with nonzero ED expenditures. The dependent variable is changed to per-member per-month ED expenditures and log-transformed. The identified high utilizers comprise 1–2% of the population through the years. The statistics of PPVs of high utilizers versus other patients are presented in Table 9. The correlation test results are shown in Table 8. All results imply that the residuals also identify a significant amount of preventable ED utilization. However, comparing the level of differences in mean statistics in Tables 7 and 9, the relationship for PPVs and residuals is weaker compared to PPRs and residuals. This generally makes sense because ED visits are more incidental than inpatient hospital visits.

**Table 9:** Potentially Preventable Emergency Department Visits (PPV) statistics of index year and next year for high utilizers versus other patients

	Index year	High Utilizers (High Residuals)	Other Patients
**Average number of PPVs in index year**	2011	1.51	1.37
	2012	3.10	1.89
	2013	3.23	1.95
	2014	2.99	1.88
**Average PPV expenditures in index year**, $	2011	1815.21	747.62
	2012	3444.05	698.43
	2013	3653.24	703.12
	2014	3986.26	664.19
**Average number of PPVs in next year**	2011	1.87	1.75
	2012	2.46	1.71
	2013	2.48	1.69
**Average PPV expenditures in next year**, $	2011	1083.07	750.32
	2012	1453.91	653.90
	2013	1495.37	647.75

Residuals and high utilizers are identified from index year. Per-member per-month emergency department expenditures is the dependent variable of the model

#### Residuals and future potentially preventable events

In Tables 7 and 9, we also show the PPR and PPV statistics for the year following the year from which we compute the residuals (index year). Although the differences in PPR and PPV utilization amounts between high utilizers and other patients are narrowed from the index year, the high-utilizers group still has significantly more PPRs and PPVs, as well as associated costs. The correlation coefficients of the next year in Table 8 also indicate a decreased but still sizable temporal correlation. Thus, the residuals are good predictors for future PPRs and PPVs as well. This finding is consistent with the strong temporal correlation of residuals we present in the previous section.

To summarize, using the 3M™ PPE software, we have demonstrated that the residuals from the models, when stratified to inpatient hospital and ED settings, are strongly associated with potentially preventable utilization of the same year as well as the next year, implying promising potential to identify impactable high utilizers.

## Discussion

In this paper, we propose a novel approach to analyze variations in Medicaid health care expenditures based on using higher-than-expected values of the residuals from health care utilization adjustment models. We conduct our analyses on a large administrative claims dataset from a state public insurance program. Our approach identifies a significant amount of unexplained health care utilization. We show that this variation could be within and between ICD-9-CM codes. Also the variation exists across the whole spectrum of health expenditures. This differs from the Medicaid high utilizers in New York City whose disease burdens were high and usage of all types of care were frequent [9]. As health care practitioners focused more on those with complicated conditions, the others with less disease burden also represents some amount of preventable health care utilization. We observed strong temporal consistency in the utilization identified by high residuals, though in Denver, Colorado people found the health care needs for high utilizers are intense but temporary [10]. As opposed to the single provider network examined in that study, our state-wide analysis provides a more comprehensive and stable picture of high utilization because we limit to the population consistently enrolled. The utilization fraction associated with high residuals is shown to be largely preventable as it produces results similar to the 3M™ PPE software. Since the PPE tools are widely used for measuring potentially preventable health care services [7], other state Medicaid programs could potentially use this approach to identify impactable high utilizers and possibly reduce inappropriate health care spending.

We specifically examined two comorbidities, essential hypertension and chronic kidney disease. In essential hypertension, a particular ICD-9-CM diagnosis code, “4019, Unspecified essential hypertension”, exhibited significant variation in expenditures. This may imply a more granular classification of hypertension is needed to stratify the health care services rendered within this diagnosis. On the other hand, patients with different stages of chronic kidney disease drove the variations in this diagnosis. Therefore in patient risk adjustment and stratification, chronic kidney disease severity should be accounted for. This will lead to more personalized and better stratified chronic kidney disease management in Medicaid.

This study is limited in several dimensions. First, though our models attempt to include a large number of exogenous variables in the administrative claims dataset, it is not comprehensive. Our models cannot account for factors (e.g. genetic variations, socioeconomic status) that are expected to be predictive of health care utilization but difficult to collect. Other important variables that contribute to health care expenditures, such as health condition severity measures, are missing. The residuals we obtain from our current model are likely to be affected by these unadjusted exogenous variables. Also, our findings are specific to the Texas Medicaid program. Second, we have not conducted case studies to follow up on specific clinical details about the high utilizers. Such an investigation could reveal more information about clinical factors contributing to the variation. Third, though the residuals prove to be highly associated with potentially preventable utilization, they do not point to specific pathways to inform policy. We recommend stakeholders to look into factors introducing variations into the model like the “unspecified essential hypertension” diagnosis with the goal of identifying modifiable sources of variation.

Our future work will try to address these limitations. We will gather additional data, such as social determinants of health and electronic medical record notes, to adjust as many exogenous factors as possible. We also plan to conduct medical record reviews with clinical and health care policy experts to identify components of care that could be addressed to reduce preventable utilization. More importantly, we will extend this exclusively data-driven approach into an iterative process [28] between health care practitioners and informatics researchers to better understand impactable health care conditions and progress toward interventions to reduce inappropriate health care utilization.

## Conclusions

To conclude the paper, we identify best-fitting risk adjustment models to account for patients’ health conditions and demographic characteristics in the Texas Medicaid program using administrative claims data. We find that populations with higher than expected expenditures are associated with more potentially preventable events. Deeper dive into the hypertension and chronic kidney disease cohorts show significant variations in expenditures within and across some diagnoses. Additionally, strong temporal consistency exists among patients with high residuals, implying possible intervention chances to reduce health care costs for this population. Overall, we believe this work presents a new way to identify impactable high utilizers in health care.

## Data Availability

The datasets analysed during the current study are not publicly available due to protected health information (PHI).
